# Multispectral Antimicrobial Blue Light (aBL) Systems for Continuous Decontamination of Food-Contact Surfaces and Environmental Matrices

**DOI:** 10.3390/foods15091550

**Published:** 2026-04-30

**Authors:** Nnabueze Darlington Nnaji, Christian Kosisochukwu Anumudu, Damion Forbes, Elroy Castelino, Taghi Miri, Helen Onyeaka

**Affiliations:** 1 School of Chemical Engineering, University of Birmingham, Birmingham B15 2TT, UKcka329@alumni.bham.ac.uk (C.K.A.);; 2 Department of Microbiology, University of Nigeria, Nsukka 410105, Nigeria

**Keywords:** antimicrobial blue light (aBL), photodynamic inactivation, visible violet–blue LED, food safety, *Escherichia coli*, *Bacillus cereus*

## Abstract

Antimicrobial blue light (aBL) within the visible violet–blue spectrum has emerged as a promising non-chemical strategy for microbial control, yet its performance across environmentally realistic matrices and surfaces remains insufficiently characterised. Here, we evaluate a continuous-exposure aBL LED system operating within the visible 407–421 nm range for its antimicrobial efficacy against *Escherichia coli* K-12 MG1655 and *Bacillus cereus* NCTC 11143 across liquid cultures, agar surfaces, and representative built-environment materials (glass and steel bar). Bacterial inactivation was quantified using culture-based enumeration and flow cytometric viability profiling. The system delivered a controlled irradiance of 0.72 mW/cm^2^ at 58 cm, corresponding to cumulative doses of 2.59–62.23 J cm^−2^ over 1–24 h of exposure. Significant, time-dependent reductions in viability were observed across all matrices relative to fluorescent-light controls, with near-complete or complete loss of recoverable cells on solid surfaces following prolonged exposure. Flow cytometric analyses revealed progressive transitions from viable to injured and dead cell populations, consistent with photodynamic inactivation mediated by endogenous photosensitiser activation and reactive oxygen species generation. These findings demonstrate that continuous visible-light aBL illumination can achieve effective multisurface microbial inactivation under moderate irradiance conditions compatible with occupied environments, supporting its translational potential as a sustainable, non-chemical decontamination strategy for healthcare, food-processing, and built environments.

## 1. Introduction

Microbial contamination of indoor environments and food-contact surfaces remains a persistent and escalating public health challenge, particularly in healthcare, food processing, and high-occupancy built environments. The persistence of pathogenic bacteria on inert surfaces in processing environments contributes significantly to cross-contamination events and healthcare-associated infections (HAIs), which continue to impose a substantial burden globally [1,2,3]. Foodborne and opportunistic pathogens, including *Escherichia coli*, *Staphylococcus aureus*, *Pseudomonas aeruginosa*, *Acinetobacter baumannii*, and *Bacillus subtilis*, can survive for extended periods on dry surfaces under ambient indoor conditions, thereby facilitating indirect transmission pathways that are difficult to control using conventional hygiene measures alone [4]. This persistence is exacerbated in modern built environments characterised by high surface-contact frequency, controlled climates, and limited natural ventilation, where microbial reservoirs can accumulate despite routine cleaning. These challenges underscore the urgent need for innovative, continuous, and non-chemical antimicrobial strategies capable of reducing environmental microbial loads across diverse matrices.

Chemical disinfectants remain the cornerstone of environmental decontamination; however, their limitations are increasingly recognised. Repeated application of chemical biocides is associated with transient efficacy, material degradation, occupational exposure risks, and growing concerns regarding the selection of tolerant or resistant microbial populations [5,6,7]. In food-processing and healthcare settings, residues from disinfectants may also compromise surface integrity or product safety, limiting their suitability for continuous use [8]. Ultraviolet (UV-C) irradiation offers high antimicrobial efficacy but is inherently constrained by photobiological hazards, including skin and ocular damage, restricting its application to unoccupied spaces and intermittent treatment cycles [9,10]. Consequently, there is growing interest in alternative light-based technologies that balance antimicrobial performance with human safety, operational continuity, and environmental sustainability.

**Figure 1:**
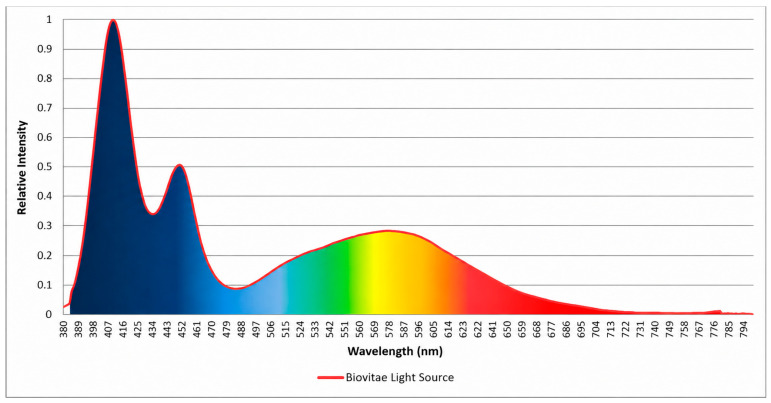
Normalised spectral emission of Biovitae light source.

Visible violet–blue light, commonly referred to as antimicrobial blue light (aBL), typically in the 400–470 nm range (Figure 1), has emerged as a promising non-chemical intervention capable of continuous deployment in occupied environments. Unlike UV irradiation, aBL exerts antimicrobial effects primarily through excitation of endogenous microbial chromophores such as porphyrins and flavins, which absorb photons and subsequently generate reactive oxygen species (ROS) that damage cellular components, including membranes, proteins, and nucleic acids [11,12,13,14]. Importantly, this mechanism reduces the likelihood of resistance development, as oxidative damage targets multiple cellular pathways simultaneously, distinguishing aBL photodynamic inactivation (PDI) from conventional antimicrobial agents that act on discrete molecular targets. Wavelengths centred around 405 nm exhibit broad-spectrum antimicrobial activity against both Gram-positive and Gram-negative bacteria without requiring exogenous photosensitisers; however, Gram-positive species are generally more susceptible, typically achieving significant reductions at lower radiant exposures, whereas higher energy doses are often required to inactivate Gram-negative organisms due to their outer membrane barrier [15,16,17,18,19].

Building on the established efficacy of single-wavelength 405 nm systems, emerging multispectral visible-light (antimicrobial white light (aWL)) approaches integrate complementary wavelengths to broaden chromophore excitation profiles, enhance antimicrobial performance across diverse microorganisms, and optimise the balance between efficacy and safety in food and environmental applications [11,20,21].

Despite growing evidence supporting the antimicrobial efficacy of aBL, significant knowledge gaps remain regarding its performance across different environmental matrices and microbial physiological states. Much of the existing literature has focused on planktonic cultures or single-surface models, limiting the translational relevance of findings to real-world conditions where microorganisms encounter heterogeneous substrates and variable exposure geometries. Furthermore, bacterial susceptibility to light-mediated inactivation is highly influenced by the microenvironment and physical characteristics of the target, which directly modulate photon penetration and ROS generation [22]. The need for systematic, multisurface evaluations under controlled yet environmentally relevant conditions has therefore been repeatedly highlighted as a critical research priority [1].

An additional complexity is that vegetative cells, as well as dormant or stress-resistant forms, e.g., bacterial spores, respond differently to exposure to aBL. A ubiquitous foodborne pathogen, *Bacillus cereus*, can serve as an example of this challenge due to its capacity to switch between the active vegetative cells and highly resilient spores that have a strong resistance to physical and chemical stress [23,24]. Although aBL is a viable method of inactivating vegetative cells, the effects on spores are weak, which requires careful interpretation of the extent and limitations of the treatment. The lack of consideration of these differences could lead to overestimation of the protective ability of light-based interventions in complex microbial ecosystems. Therefore, the combination of viability-based analytical techniques, including flow cytometry, with conventional culture methods is becoming increasingly accepted as a criterion of the proper characterisation of antimicrobial results [25].

In addition to efficacy, photothermal effects and environmental safety of aBL systems should be given serious consideration in practice. Although aBL could be deemed less damaging as compared to UV irradiation, the long-term exposure to a high dose of irradiance may lead to temperature rises that would indirectly result in microbial stress or inactivation [26,27]. It is thus important to differentiate photochemical and photothermal effects when interpreting the mechanism and to achieve reproducibility in different settings. The ability to regulate temperature and dose can be important when testing new technologies of aBL, particularly in closed volumes where the heat loss can be reduced to a minimum [26]. These considerations indicate the need to have experimental designs that are strictly regulated and reflect both microbiological and environmental reality.

It is in this context that this study presents a detailed experimental study on an aBL-based antimicrobial system in liquid culture, agar culture and realistic built-environment surfaces, e.g., steel and glass. The study aims to close the gap in knowledge on multisurface efficacy of aBL-based systems using both culture-based approaches and flow cytometric viability measurements to gauge microbial physiological response to their application. This will guide practical deployment considerations. The study utilised *Escherichia coli* K-12 MG1655 and *Bacillus cereus* NCTC 11143 as indicator/test organisms. *E. coli* K-12 MG1655, a well-characterised laboratory model organism, was selected due to its genetic stability and reproducibility [28], and its use enables controlled evaluation of fundamental photoinactivation responses. *B. cereus* NCTC 11143 was selected as a well-characterised reference strain representative of foodborne, Gram-positive bacteria, widely used in environmental and food microbiology to provide reproducible and comparable antimicrobial efficacy assessments [29]. *E. coli* and *B. cereus* are industrially important bacterial species, the environmental survival of which is well-documented in causing food safety and health hazards to the population [1,4,30]. This study focuses on planktonic and surface-associated vegetative cells as an initial step toward understanding multisurface aBL efficacy. The results of this multisystem approach are expected to contribute to the further development of the knowledge about aBL technologies as a scalable, non-chemical method of environmental decontamination in modern built environments.

## 2. Materials and Methods

### 2.1. Bacterial Strains and Culture Preparation

The bacterial strains used in this study were *Escherichia coli* K-12 MG1655 and *Bacillus cereus* NCTC 11143, selected to represent Gram-negative and Gram-positive bacteria of relevance to environmental persistence, food safety, and antimicrobial efficacy testing.

Lyophilised cultures of both strains were obtained from the microbial culture collection of the Biochemistry Laboratory, School of Chemical Engineering, University of Birmingham. Cultures were revived by aseptic transfer of a single bead into sterile nutrient broth (Oxoid Ltd., Basingstoke, UK). The cultures were incubated at 37 °C, orbitally shaken at 150 rpm for 18 h to enable resuscitation and active growth. Purity of culture was confirmed by streaking on nutrient agar (Oxoid Ltd., Basingstoke, UK) and incubating at 37 °C for 24 h and 48 h for *E. coli* and *B. cereus* respectively, with colony morphology examined to confirm the absence of contamination. To control variability in all experiments, freshly prepared overnight cultures were used as starter cultures to reduce variability due to long periods of growth in stationary phase.

To achieve uniformity and reproducibility of physiological data with respect to experimental replicates, bacterial cultures were standardised to a specified growth phase and cell density before exposure to light. Subcultures were prepared by inoculating fresh nutrient broth with overnight cultures and incubated under the same conditions until mid-logarithmic growth was achieved. The growth of the bacteria was observed spectrophotometrically at 600 nm and the cultures were corrected with sterile nutrient broth to attain the desired optical density. An OD_600_ of 0.2 corresponding to approximately 1 × 10^6^ CFU mL^−1^ (6 log CFU mL^−1^) was employed as the working concentration of inoculum in all further experiments, in nutrient broth, on agar plates and in surface contamination assays.

### 2.2. Experimental Setup and Light Exposure Conditions

**Figure 2:**
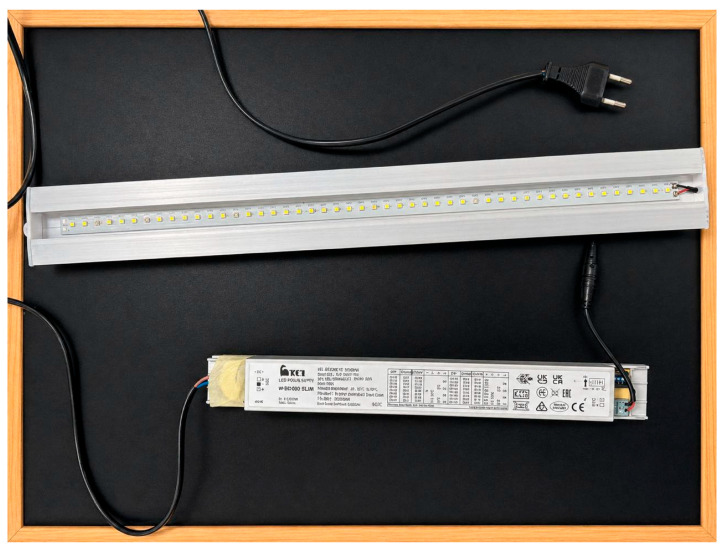
The aBL LED source.

The antimicrobial light source used in this study was provided by Nextsense S.r.l. Salerno, Italy, the developer of the patented Biovitae^®^ visible-light technology. Normalised spectral emission of the light source is presented in Figure 1. The lighting system consists of 50 high-efficiency white and blue LEDs (3535 package type) mounted on a 1.2 mm metal-core printed circuit board (MCPCB) with an integrated aluminium heat sink (see Figure 2). The light source is characterised by a continuous, polychromatic visible emission spanning 380–780 nm. The spectral power distribution (SPD) exhibits distinct spectral features in the violet–blue region, with components centred at 407 nm, 416 nm, and 421 nm, which represent the principal antimicrobial emission band. In addition, a secondary emission component in the royal-blue region at approximately 450 nm is present, contributing to phosphor excitation and the resulting broadband spectral conversion that produces general white-light illumination. The device does not emit, nor incorporate, any UV-emitting components and provides white-light illumination with a correlated colour temperature (CCT) of 8000 K.

Unlike most aBL systems reported in the literature, which rely on narrow-band monochromatic emission, the antimicrobial white-light (aWL) source used in this study represents an evolution of aBL technology in which the antimicrobial violet–blue emission is embedded within a broader white-light visible spectrum. The device therefore delivers simultaneous illumination and antimicrobial activity through a continuous polychromatic emission profile. Although the radiant dose is reported with reference to the 400–420 nm spectral band, biological samples were exposed to the full visible emission of the aWL source, resulting in a total radiant exposure that includes contributions from the entire visible spectrum. The referenced 400–420 nm band corresponds to the dominant absorption (Soret) region of endogenous bacterial porphyrins, while longer wavelengths contribute comparatively less due to weaker chromophore absorption [31].

**Figure 3:**
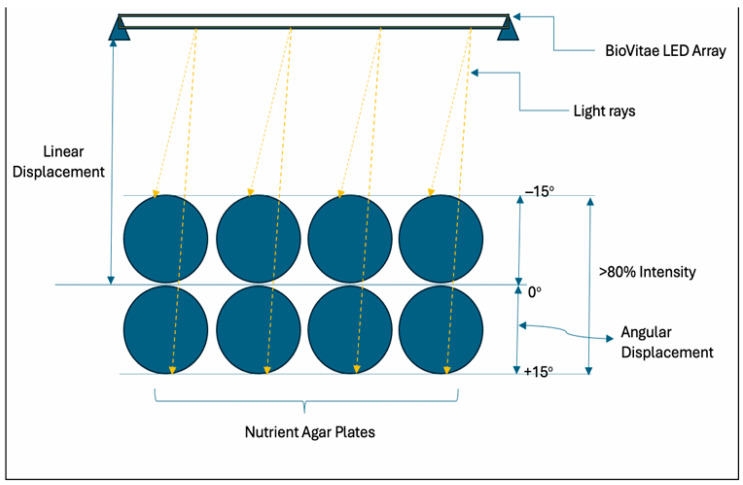
The experiment setup for agar surface exposure under the LED array (adapted from the manufacturer’s technical specifications).

The aBL LED source provides peak reference irradiances in the spectral range of 400–420 nm of 0.936 mW/cm^2^ at 50 cm and 3.742 mW/cm^2^ at 25 cm. The luminaire exhibited a nominal beam angle of 55° (FWHM), with relative luminance intensity exceeding 80% within ±15° from the optical axis and progressively decreasing beyond ±27.5°. This guided the experimental setup (see Figure 3). Irradiance values at experimental distances were calculated using the inverse square law:

I_1_/I_2_ = (r_2_^2^/r_1_^2^) (1)

where I_1_ is the reference irradiance at distance r_1_ and I_2_ is the irradiance at distance r_2_. The delivered light dose was calculated as:

Dose (J cm^−2^) = Irradiance (W cm^−2^) × Exposure time (s) (2)

All experiments were conducted within Class II biological safety cabinets (Gelaire Safety Cabinet BSB 3 S, The Baker Company, Sanford, ME, USA). Prior to experimentation, cabinet surfaces were disinfected with 70% (*v*/*v*) ethanol (Fisher Scientific UK Ltd., Loughborough, UK), followed by UV irradiation for 30 min. Cabinets were then ventilated for 15 min before use.

Temperature monitoring was performed using an Extech^®^ Instruments SDL200 four-channel temperature data logger (Extech Instruments, FLIR Systems, Wilsonville, OR, USA). Thermocouples were positioned to record internal test cabinet and control cabinet temperatures throughout each experiment, including exposure periods of up to 24 h.

### 2.3. Broth, Agar, and Surface Exposure Assays

Broth cultures of *E. coli* and *B. cereus* were adjusted to an optical density of 0.2. Aliquots (30 mL) corresponding to a broth depth of 2.1 cm in sterile glass beakers were transferred into sterile glass beakers containing sterile magnetic stirring rods and exposed to continuous illumination under the light source with constant agitation provided by magnetic stirring. This consistent geometry was maintained across all treatments to ensure comparability between treatments. Nutrient agar plates were inoculated with 100 µL of bacterial suspension adjusted to an optical density of 0.2 and spread evenly across the surface. Glass slides and steel bar coupons were sterilised by autoclaving and marked to define a consistent exposure area (25 × 75 mm), then inoculated with 100 µL of bacterial suspension evenly distributed across the defined area.

**Figure 4:**
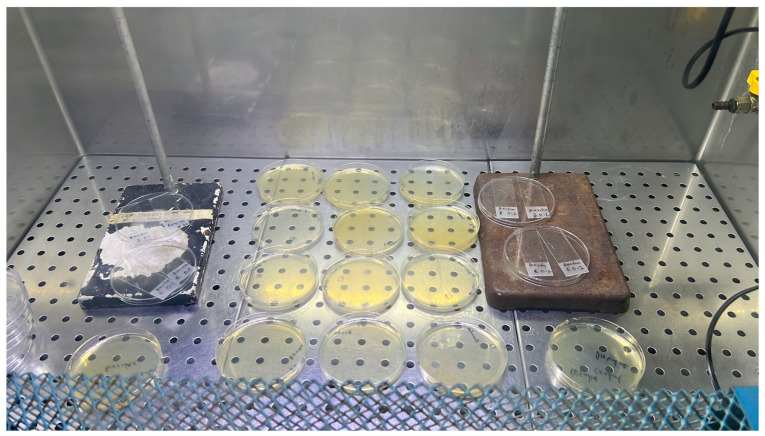
Nutrient agar plates and glass slides under exposure to aBL LED source.

**Figure 5:**
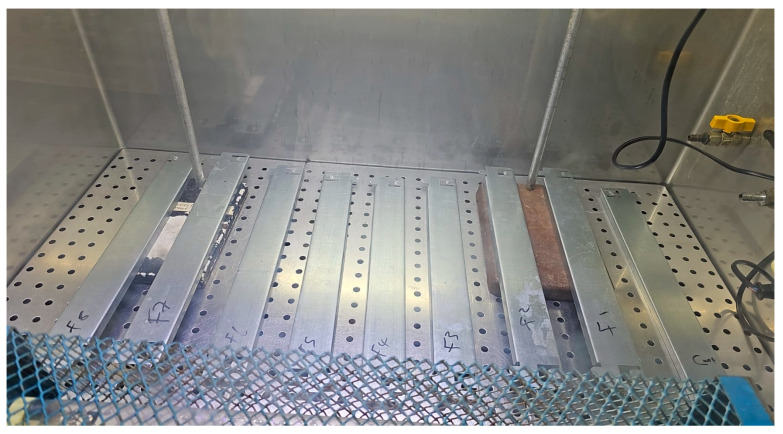
Steel bar surface treatment using aBL LED source.

All the inoculated samples were subjected to the aBL LED source or fluorescent-light control at a constant distance of 58 cm for 1 h, 3 h, 6 h, or 24 h. A fluorescent-light control was selected to represent realistic indoor lighting conditions. Agar plates and glass slide exposure configurations are shown in Figure 4, while the steel bar surface treatment setup is shown in Figure 5. For each condition, all experiments were performed in triplicate for each bacterial strain.

Following exposure, microbial recovery and enumeration were performed according to sample type. Agar plates were incubated directly at 37 °C for 24 h. Broth samples were serially diluted in sterile phosphate-buffered saline (PBS: Thermo Fisher Scientific, Waltham, MA, USA), plated onto nutrient agar, and incubated at 37 °C for 24 h. Steel bar coupons were sampled by swabbing the defined exposure area using sterile swabs, which were eluted in PBS prior to serial dilution and plating. Microbial inactivation was quantified as log_10_ CFU reduction relative to corresponding fluorescent-light control samples.

### 2.4. Flow Cytometric Analysis

Following light exposure, broth cultures of *E. coli* and *B. cereus* designated for flow cytometric analysis were harvested by centrifugation at 5000 rpm and washed twice in sterile PBS. Cell pellets were resuspended in PBS and stained with propidium iodide (Sigma-Aldrich, St. Louis, MO, USA) and DiBAC_4_(3) (bis-(1,3-dibutylbarbituric acid) trimethine oxonol) (Thermo Fisher Scientific, Waltham, MA, USA). Stained samples were incubated at 37 °C for 5 min prior to analysis. Flow cytometric measurements were performed using a BD Accuri C6 Plus flow cytometer (BD Biosciences, Ann Arbor, MI, USA). For each sample, 25,000 events were acquired at a flow rate of 1000–4000 events s^−1^. Data analysis was conducted using predefined gating strategies derived from live and dead *E. coli* and *B. cereus* populations, enabling discrimination of live, injured, and dead cells.

### 2.5. Statistical Analysis

Data were organised and pre-processed using Microsoft^®^ 365 Excel (Microsoft Corporation, Redmond, WA, USA; Version 2402). Colony-forming unit counts were log_10_-transformed prior to statistical analysis, and irradiance and dose values were calculated within the same software. Results are expressed as mean ± standard deviation. Statistical analyses were conducted using SPSS Statistics version 26 (IBM Corp., Armonk, NY, USA), with differences between treatment groups evaluated at the 95% confidence level using analysis of variance (ANOVA) followed by least significant difference (LSD) post hoc tests.

## 3. Results

Table 1 summarises the irradiance and corresponding energy doses delivered by the LED system at a fixed distance of 58 cm. At this distance, the measured irradiance in the spectral range of 400–420 nm was 0.72 mW/cm^2^, yielding cumulative doses of 2.59 J/cm^2^, 7.78 J/cm^2^, 15.56 J/cm^2^, and 62.23 J/cm^2^ after 1, 3, 6, and 24 h of continuous exposure, respectively. As expected, delivered dose increased proportionally with exposure duration at constant irradiance.

Temperature monitoring indicated stable operating conditions for both lighting systems throughout the experiments (Table 2). Under aBL LED illumination, recorded temperatures ranged from 28.6 to 39.6 °C, with a mean value of 35.8 ± 0.9 °C. Fluorescent-light conditions exhibited temperatures between 26.3 and 32.5 °C, with a mean of 28.1 ± 0.8 °C.

### 3.1. Photoinactivation of Bacteria on Agar Surfaces

**Figure 6:**
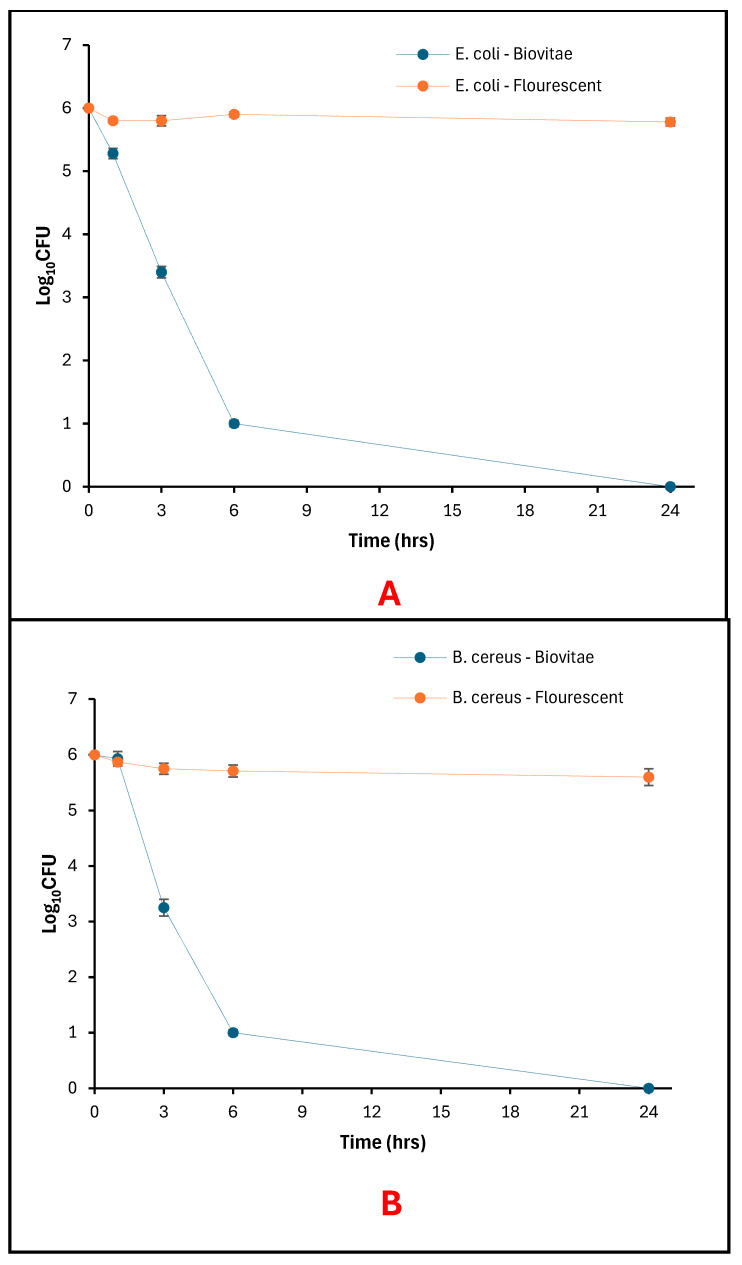
Bacteria viability on Agar as a function of exposure time: (**A**) surviving *E. coli* populations; (**B**) Surviving *B. cereus* populations.

The aBL LED exposure resulted in a time-dependent reduction in bacterial viability on agar surfaces for both *E. coli* and *B. cereus* under aBL LED illumination, while no comparable reduction was observed under fluorescent-light conditions (Figure 6A,B).

**Figure 7:**
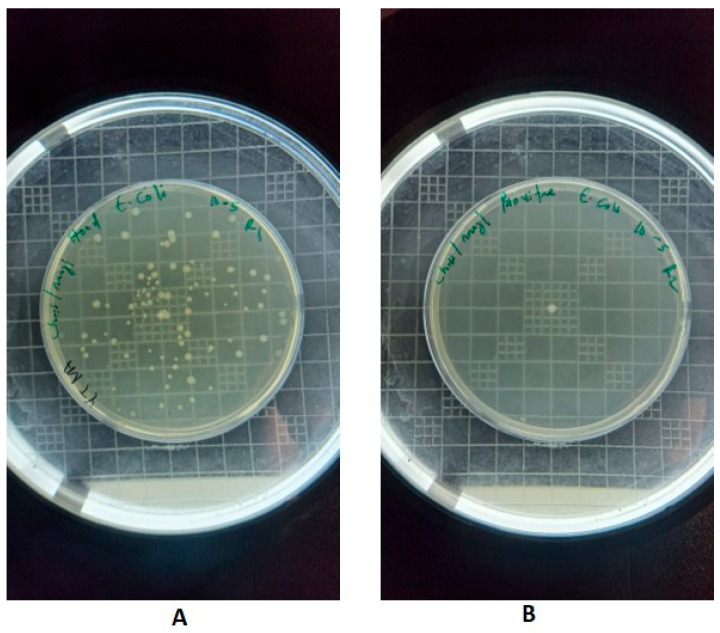
(**A**) *E. coli* following treatment with fluorescent light, (**B**) *E. coli* following treatment with aBL LED source. Images shown correspond to samples after 24 h of exposure under the respective lighting conditions.

For *E. coli*, the initial viable count at 0 h was 6 log_10_ CFU for both the aBL LED and fluorescent-light control samples. Following exposure to the evaluated LED array, viable counts decreased to 5.28 log_10_ CFU after 1 h, 3.40 log_10_ CFU after 3 h, and 1.00 log_10_ CFU after 6 h, with no detectable colonies observed after 24 h (Figure 7). In contrast, fluorescent-light controls maintained relatively stable viable counts across all time points, ranging from 5.78 to 5.90 log_10_ CFU over the 24 h period (Figure 6A).

**Figure 8:**
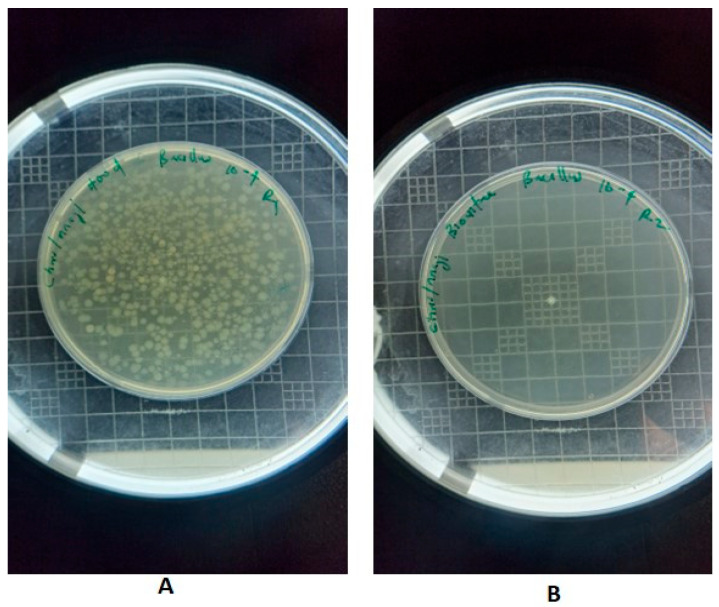
(**A**) *B. cereus* following treatment with fluorescent light, (**B**) *B. cereus* following treatment with aBL LED source. Images shown correspond to samples after 24 h of exposure under the respective lighting conditions.

Similarly, *B. cereus* exhibited an initial viable count of 6 log_10_ CFU under both lighting conditions at 0 h. Under the evaluated LED, viable counts were reduced to 5.93 log_10_ CFU after 1 h, 3.25 log_10_ CFU after 3 h, and 1.00 log_10_ CFU after 6 h, with no detectable colonies at 24 h (Figure 8). Fluorescent-light control samples showed minimal variation in viable counts, remaining between 5.60 and 5.87 log_10_ CFU throughout the exposure period (Figure 6B).

### 3.2. Photoinactivation of Bacteria in Broth Cultures

**Figure 9:**
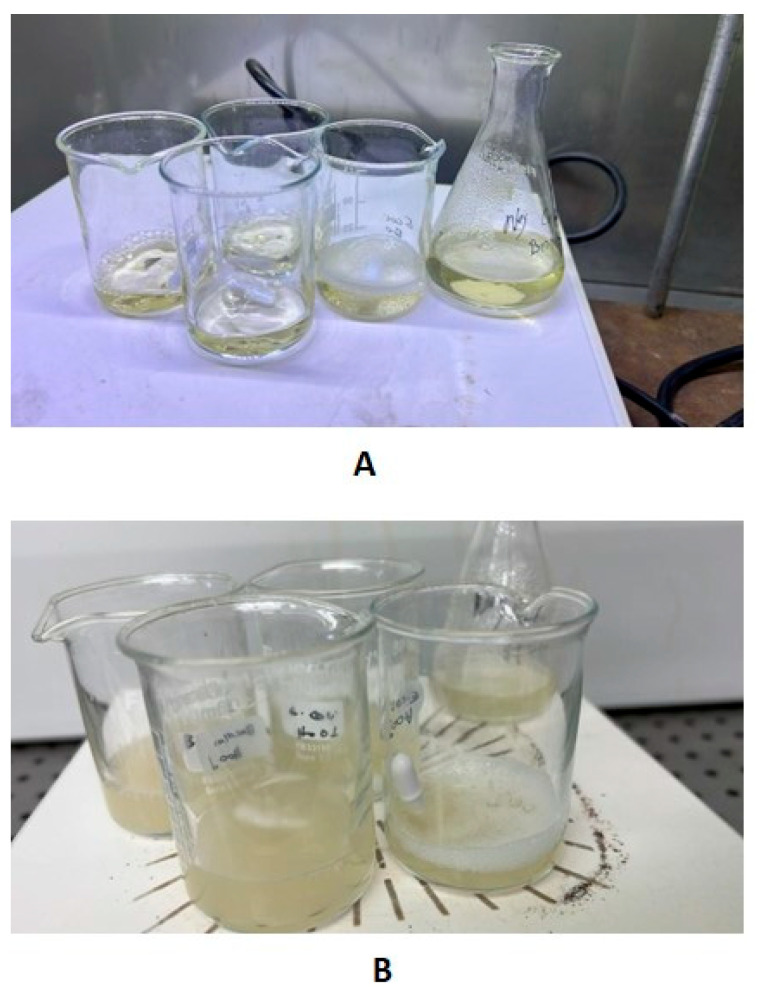
(**A**) Broth under aBL LED source, (**B**) Broth under fluorescent light. Images shown correspond to samples after 24 h of exposure under the respective lighting conditions.

**Figure 10:**
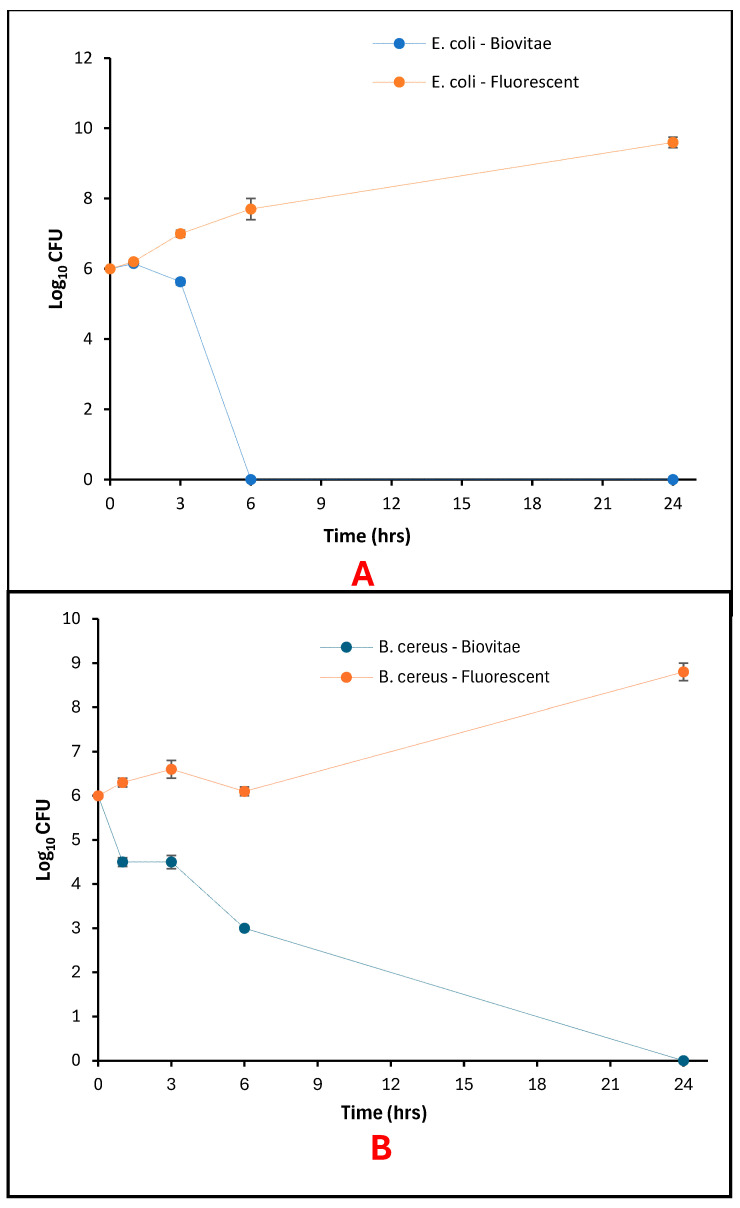
Bacteria viability in Broth as a function of exposure time: (**A**) surviving *E. coli* populations; (**B**) Surviving *B. cereus* populations.

Broth cultures incubated under fluorescent light had a visible turbidity after 24 h, but under the aBL LED, broths were observed to be visually clear and similar to uninoculated nutrient broth (Figure 9). The viability of bacterial cultures in broth samples declined with time under the aBL LED for both *E. coli* and *B. cereus*, but the viable counts remained constant or increased during the same time frame under the fluorescent-light controls (Figure 10A,B).

For *E. coli*, initial counts were 6 log_10_ CFU at 0 h under both lighting conditions. The viable counts under the aBL LED were 6.15 log_10_ CFU after 1 h and 5.63 log_10_ CFU after 3 h, with no observed colonies after 6 h and 24 h. Conversely, the fluorescent-light controls exhibited gradual increments in viable counts with 7.7 log_10_ CFU at 6 h and 9.6 log_10_ CFU at 24 h (Figure 10A).

For *B. cereus*, initial counts were also 6 log_10_ CFU at 0 h. Viable counts under the aBL LED were 4.5 log_10_ CFU at 1 h and remained at this level at 3 h, followed by a reduction to 3 log_10_ CFU at 6 h, and no colonies were detected at 24 h. The control with fluorescent lights had a higher viable count with a starting count of 6.3 log_10_ CFU at 1 h to 8.8 log_10_ CFU at 24 h (Figure 10B).

### 3.3. Photoinactivation of Bacteria on Glass Surfaces

**Figure 11:**
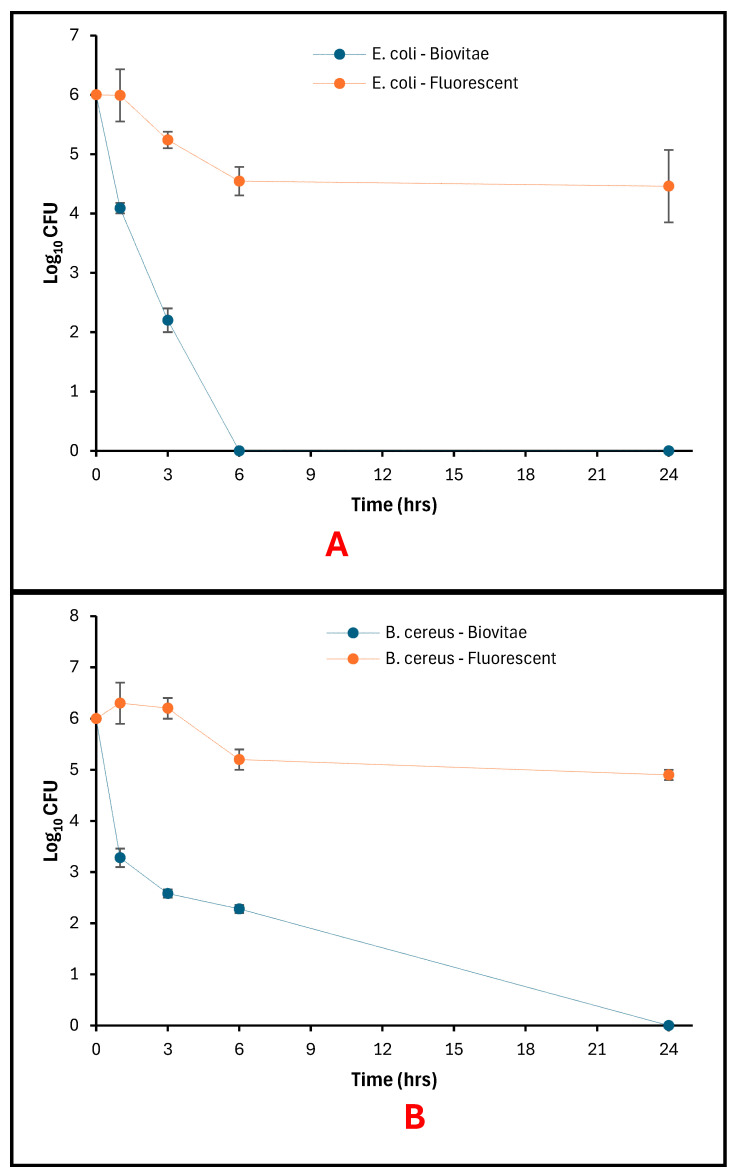
Bacteria viability on Glass as a function of exposure time: (**A**) surviving *E. coli* populations; (**B**) Surviving *B. cereus* populations.

Bacterial recovery from glass surfaces decreased over time under the evaluated LED light for both *E. coli* and *B. cereus*, while viable counts remained higher under fluorescent-light conditions (Figure 11A,B).

For *E. coli*, initial counts were 6 log_10_ CFU at 0 h under both lighting conditions. Under the evaluated LED light, viable counts declined to 4.09 log_10_ CFU at 1 h and 2.20 log_10_ CFU at 3 h, with no detectable colonies recovered at 6 h or 24 h. Fluorescent-light controls showed a more gradual decrease, with viable counts of 4.55 log_10_ CFU at 6 h and 4.46 log_10_ CFU at 24 h (Figure 11A).

For *B. cereus*, initial viable counts were 6 log_10_ CFU at 0 h. Following exposure to the evaluated LED light, viable counts were 3.28 log_10_ CFU at 1 h, 2.58 log_10_ CFU at 3 h, and 2.28 log_10_ CFU at 6 h, with no detectable colonies at 24 h. Under fluorescent-light conditions, viable counts remained between 4.9 and 6.3 log_10_ CFU throughout the exposure period (Figure 11B).

### 3.4. Photoinactivation of Bacteria on Steel Bar Surfaces

**Figure 12:**
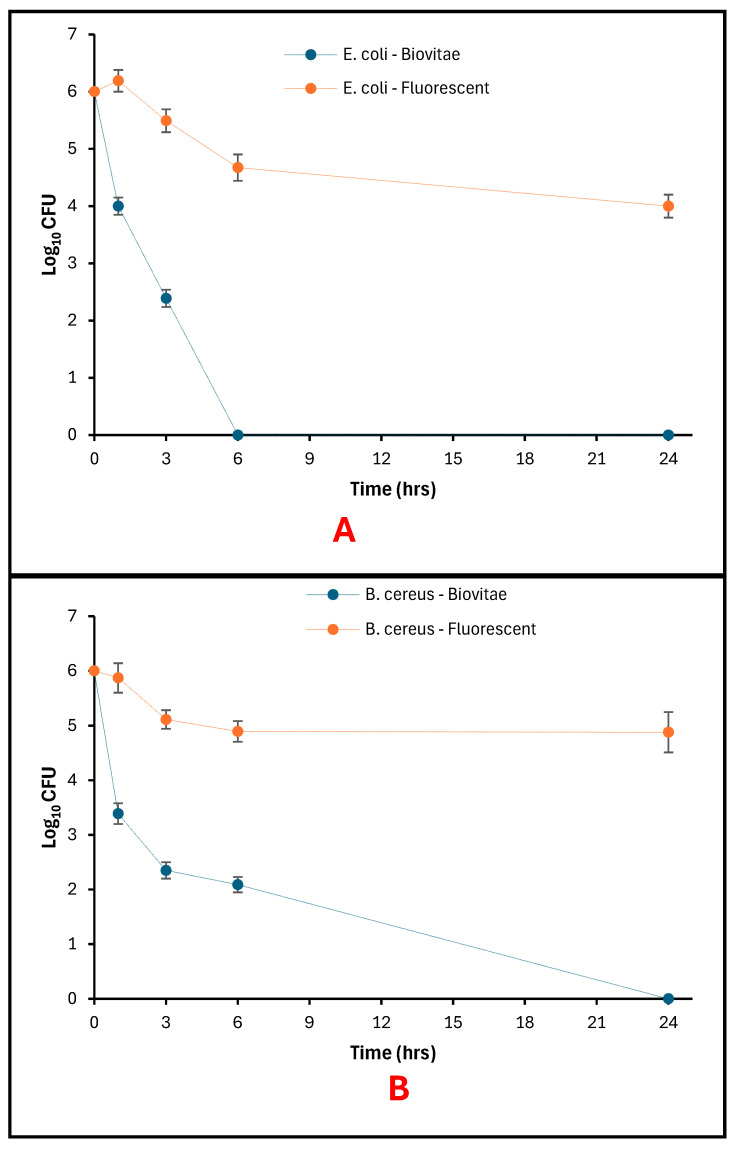
Bacteria viability on Steel Bar as a function of exposure time: (**A**) surviving *E. coli* populations; (**B**) Surviving *B. cereus* populations.

With the aBL LED treatment, bacterial recovery of stainless-steel bars decreased with exposure time of both *E. coli* and *B. cereus*, and at the same time, greater viable counts were maintained under fluorescent-light conditions (Figure 12A,B).

For *E. coli*, viable counts at 0 h were 6 log_10_ CFU under both lighting conditions. With the aBL LED, the number of counts reduced to 4.00 log_10_ CFU at 1 h and 2.39 log_10_ CFU at 3 h, with no colonies recovered at 6 h or 24 h. Fluorescent-light controls had more recoverable counts, with 4.67 log_10_ CFU at 6 h and 4.00 log_10_ CFU at 24 h (Figure 12A).

For *B. cereus*, initial counts were 6 log_10_ CFU at 0 h. The viable counts under the aBL LED were 3.39 log_10_ CFU at 1 h, 2.35 log_10_ CFU at 3 h, and 2.09 log_10_ CFU at 6 h, with no colonies detected at 24 h. Fluorescent-light controls maintained viable counts ranging from 4.88 to 5.87 log_10_ CFU throughout the exposure period (Figure 12B).

The post hoc analysis of all surfaces’ treatment revealed statistically significant differences between treatments with each type of surface with no differences at baseline but clear separation in the subsequent analysis; the treatments with fluorescent light were always ranked higher and the treatments with the aBL LED light were always ranked lower since the light inactivated the bacteria (Table 3).

### 3.5. Flow Cytometric Assessment of E. coli Viability in Broth Cultures

**Figure 13:**
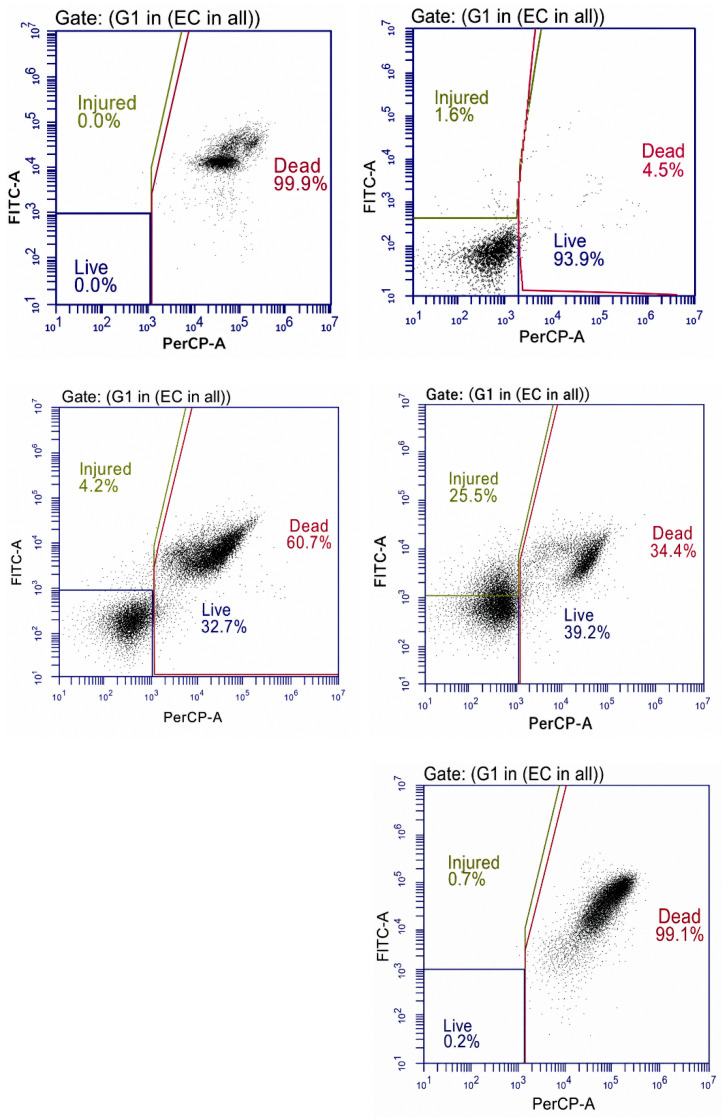
Flow cytometric assessment of *E. coli* and *B. cereus* viability in broth following aBL LED exposure. Left panels depict *E. coli* after 3 h and 6 h of exposure, whereas right panels depict *B. cereus* after 1 h, 6 h, and 24 h of exposure. Live (FITC^−^/PerCP^−^), injured (FITC^+^/PerCP^−^), and dead (FITC^+^/PerCP^+^) populations are shown.

Flow cytometric analysis further confirmed rapid loss of *E. coli* viability in broth under the evaluated LED light. After 3 h of treatment, 60.7% of cells were classified as dead, with 32.7% remaining viable and 4.2% injured (Figure 13). By 6 h of exposure, the *E. coli* population consisted almost entirely of dead cells (99.9%), with no detectable live or injured cells, indicating near-complete photoinactivation in liquid culture.

Flow cytometric analysis of *B. cereus* broth cultures revealed a time-dependent loss of viability under aBL LED exposure (Figure 13). After 1 h, most cells remained viable (93.9%), with only minor injured (1.6%) and dead (4.5%) populations detected. By 6 h, a pronounced shift toward injured (25.5%) and dead (34.4%) subpopulations was evident, accompanied by a reduction in live cells to 39.2%. After 24 h of exposure, the population was almost entirely non-viable, with 99.1% of cells classified as dead and only 0.2% remaining viable.

## 4. Discussion

The present study supports that aBL LED enriched in the 400–420 nm violet–blue spectral region can exert significant antimicrobial activity against *E. coli* and *B. cereus* across multiple matrices, including agar surfaces, broth cultures, glass, and steel bar surfaces. Notably, the antimicrobial effects here were at a quantified irradiance in the spectral range of 400–420 nm of 0.72 mW/cm^2^ at a working distance of 58 cm, which is equivalent to cumulative doses of about 2.6, 7.8, 15.6 and 62.2 J/cm^2^ at 1, 3, 6 and 24 h of constant exposure respectively. This indicates that the observed inactivation tendencies were realised in a prescribed and reproducible dose paradigm and that a dose–time relationship was in keeping with PDI processes. These results are consistent with an accumulating amount of evidence that aBL LED in this spectral range can be an effective non-chemical disinfection modality by PDI, where the photoexcitation of endogenous bacterial chromophores is triggered to generate ROS [19,32]. The aBL-LED-mediated antimicrobial effects have been reported against a range of bacterial species and biofilms, with violet (≈405 nm) wavelengths generally exhibiting the highest inactivation efficacy due to strong absorption by endogenous porphyrins and flavins within bacterial cells [16,17,19]. This mechanism is also supported by the current findings, which shows similar log-level reductions with various environmental models without external photosensitisers, and this further supports the practical value of aBL LED in environmental decontamination. These trends were supported by LSD post hoc analysis, which showed no treatment differences at baseline but significant separation at all subsequent time points, with surviving bacteria in fluorescent treatments consistently grouping higher and aBL LED treatments lower across all tested surfaces.

While the control fluorescent lighting may contain minor components within the blue region of the spectrum, its irradiance within the antimicrobial 400–420 nm range is substantially lower than that of the aBL LED system used in this study. This is supported by the absence of any significant reduction in bacterial viability under fluorescent-light conditions across all matrices, indicating that its antimicrobial contribution was negligible under the experimental conditions.

On nutrient agar surfaces, both *E. coli* and *B. cereus* had distinct decreasing recoverable viable counts with aBL LED exposure, which dropped to below detectable levels after 24 h, and no changes in viable counts with fluorescent-light controls. Since irradiance was held constant, cumulative dose was directly proportional to exposure time, meaning that the progressive decreases on agar surfaces are directly proportional to the cumulative delivered energy dose and not to varying exposure conditions. The observations are in line with the results of other aBL LED systems, in which violet wavelengths cause cumulative oxidative damage leading to cell death and interference with vital cellular processes, including membrane integrity and metabolism [33]. Equally, experiments of biofilm inactivation have shown reductions up to approximately 6.8 log_10_ CFU of *Pseudomonas fluorescens* and 3.7 log_10_ CFU of *Staphylococcus epidermidis* with violet (400 nm) LED irradiation after several hours, which highlights a general susceptibility of Gram-negative and Gram-positive bacteria to this antimicrobial modality [32]. Although the magnitude of the log-reduction and the doses needed to produce a time-dependent inactivation are dependent on the organism and experimental conditions, the general trend of the time-dependent inactivation in the present study corroborates the ROS-mediated photoinactivation reported by D’Agostini et al. [11].

The results from the broth cultures also depict the variation in the dynamics of aBL LED inactivation of liquid systems and surface exposures. The populations of *E. coli* under aBL LED exposure went to non-detectable levels after 6 h in nutrient broth, whereas in fluorescent controls, the viable counts grew over time. This result is likely due to the attenuation of light in the broth medium and inherent bacterial resistance mechanisms. In prior studies, effective inactivation of planktonic bacteria in suspension using aBL LED required substantially higher doses (often exceeding hundreds of J/cm^2^) and higher irradiances. For example, *E. coli* suspended in phosphate-buffered saline has been shown to require doses up to ~450 J/cm^2^ at 405 nm to achieve >6 log_10_ inactivation, while *S. aureus* required lower doses (~100–175 J/cm^2^) under similar conditions [34]. The reduced irradiance (0.72 mW/cm^2^) and optical density of nutrient broth in the current study were probably the reason why photon penetration and ROS formation were restrained, leading to the relatively low initial reductions and growth rebound. In comparison, the highest cumulative dose administered in the current study in 24 h (62.23 J/cm^2^) was less than most high-irradiance laboratory protocols, which is likely one reason why the inactivation kinetics in nutrient broth were slower in the present study than in many laboratory protocols. These findings indicate that the optical nature of the medium, irradiance, and dosage of photoinactivation, especially in opaque or nutrient-containing liquid, in which light scattering and absorption diminish the impact of antimicrobial activity, are important.

Glass and steel bar surface exposures showed strong and sustained decreases in bacterial viability under aBL LED. Bacterial survival on these inert surfaces decreased quickly with *E. coli* and *B. cereus* going below the detection limits under aBL LED exposure from 6–24 h, but fluorescent-light controls had a higher viability across the board. It is noteworthy that these decreases were obtained at moderate irradiance and cumulative dose levels lower than those typically reported in high-intensity laboratory systems, indicating that significant antimicrobial effects could be obtained in realistic continuous-exposure lighting in occupied conditions of indoor settings. These findings have a major implication because glass and steel are typical food-contact and built-environment surfaces that have been known to harbour endemic microbial contaminants and are also known to be sources of cross-contamination [4]. Bacterial inactivation on surfaces by aBL LED has been reported in other studies, including significant log-level reductions achieved with 405 nm LED systems at moderate irradiances (~0.5 mW/cm^2^) across extended exposure periods [19]. Moreover, the effects of surface reflectance and secondary illumination can be used to improve the local distribution of photons, and this may lead to a more effective photoinactivation than in liquid media where attenuation is more significant. The uniformity of surface outcomes of both Gram-negative and Gram-positive organisms indicates that the aBL LED photoinactivation approach may be a universal method of managing environmental contamination, which is especially applicable to infection-prone environments, e.g., hospitals and food-processing plants.

The robustness of the differences in the inactivation of *E. coli* and *B. cereus* by the aBL is further supported by consistently large effect sizes (η^2^ = 0.978–0.996) observed during the 1–24 h exposure experiments on all tested surfaces (agar, broth, glass and steel bar). These very high η^2^ values indicate that treatment conditions accounted for the variance in bacterial inactivation during the exposure period, thereby confirming the strong biological relevance of the observed effects.

The flow cytometric analysis gave a deeper insight into the physiological changes that were caused by the aBL LED. In the case of *E. coli* in broth, a rising percentage of injured and dead cells was recorded following initial exposure with almost total killing occurring by 6 h. *B. cereus* also exhibited a transition to injured and dead populations over time with nearly all cells becoming non-viable by 24 h. This trend promotes the mechanism behind the formation of ROS and the multidimensional damage of cells, including membranes, proteins, and nucleic acids [35]. During aBL LED treatments, endogenous porphyrins are photoexcited in bacterial cells to generate singlet oxygen and other ROS that subsequently damage important cellular components, causing cellular viability loss [19]. Flow cytometry allows distinguishing between live, injured and dead subpopulations beyond the simple culturability, providing an understanding of the sublethal effects and the transition to the viable but non-culturable state, which would not be observed using CFU assays alone. Other studies have also used viability dyes and cytometric methods to verify ROS-induced damage under aBL LED, which also confirms the mechanistic interpretations presented here [33].

One of the crucial control factors in the photoinactivation experiments is temperature which is also a significant interpretive constraint in the current study. Despite the constant monitoring of temperature, the average temperature varied between the conditions of light exposure, with the aBL LED exposure at 35.8 ± 0.9 °C, and the fluorescent controls being 28.1 ± 0.8 °C. Although these values are in the mesophilic range of growth and below temperatures commonly used to indicate direct thermal inactivation, thermal equivalence is absent, so temperature-related influences on growth dynamics, metabolic rate, and stress responses could be one of the sources of the observed differences in viable counts, especially with longer (24 h) exposures. Therefore, photodynamic mechanisms cannot be considered as the sole cause of the antimicrobial effects in this case. Instead, the findings can best be viewed as primarily light-driven, with potential secondary contributions from temperature-related effects. Future studies incorporating active temperature matching or thermal-only controls will be necessary to fully decouple these effects.

The relative ineffectiveness of the aBL LED inactivation of test organisms in nutrient broth when compared to surfaces and agar points out another practical reality: the effectiveness of the aBL LED is highly contingent on the optical scenario and the characteristics of the media. The turbidity and absorption of the organic components of nutrient-rich media significantly lower the light penetration to restrict the production of ROS throughout the bacterial suspension. This has been demonstrated as equally limiting to opaque or complex food matrices that become a significant barrier to the aBL LED penetration of antimicrobials and often require an increase in irradiance and cumulative dose to induce significant inactivation [36]. This is especially applicable to practical applications that are directed at food safety where liquid food products (e.g., milk, juices) and food residues may hinder the transmission of light. Synergistic approaches involving aBL LED with other interventions (e.g., mild heating, pulsed light or mild sanitiser application) can be required in such situations so that desired microbial reductions can be achieved without affecting the quality of the food.

The other notable attribute of the aBL LED antimicrobial strategies is that they possess a safety profile unlike the conventional ultraviolet disinfection. Although UV-C is an incredibly potent instrument of microbial inactivation, photobiological hazards in human tissues and material degradation limit its use to empty spaces or intermittent use [37]. The aBL LED of the violet–blue range, in comparison, does not fall within the UV-C range and is found to be safer to continuous human habitation, extending its application to the real world such as in hospitals, food-processing facilities, and other public spaces without extensive protective measures. This safety advantage, combined with the antimicrobial efficacy demonstrated at practical exposure doses, positions aBL LED photoinactivation as a promising supplemental technology for infection control and environmental hygiene.

However, the limitation of decreased performance in opaque liquid matrices indicates the necessity of further optimisation and validation. The aBL LED antimicrobial effects are dose-dependent, implying that accuracy in calibration of irradiance, exposure time and the positioning of the system are critical to efficacy, especially in heterogeneous environments. Research has also shown that not all bacteria species are equally susceptible to aBL LED antimicrobial treatment, and species-specific challenge studies are therefore important when assessing aBL LED technologies [32,38]. The immediate measurement of irradiance and determination of delivered dose at the working distance are strengths of the current study, as they allow the reproducibility and comparison of other aBL LED antimicrobial systems. Nevertheless, this work should be followed by spatial dose mapping over exposure fields in the future to further improve dose response modelling.

## 5. Conclusions

This study supports that continuous exposure to aBL LED enriched in the 400–420 nm violet–blue spectral region induces a robust, time-dependent reduction in *E. coli* and *B. cereus* viability across agar, broth, glass, and steel bar surfaces. In all tested matrices, illumination from the aBL LED source consistently resulted in significant microbial inactivation, frequently leading to complete loss of recoverable cells within 6–24 h. Flow cytometric analyses corroborated these findings by revealing progressive cellular injury and near-complete loss of viability in liquid cultures. However, because mean exposure temperatures differed between treatment and control conditions, the contribution of temperature-related effects cannot be completely excluded.

The principal novelty of this work is to show the successful aBL-LED-mediated photoinactivation of both liquid and surface-associated bacteria, using a multispectral visible-light source without ultraviolet emission under continuous exposure conditions that are similar to real-life applications. This paper identifies cumulative exposure time as an important criterion of antimicrobial activity independent of ultraviolet radiation or chemical disinfectants.

From an applied perspective, the results justify the implementation of aBL LED antimicrobial lighting as a safe, 24 h operating and energy-efficient method of bacterial load reduction of the environment in occupied indoor spaces. In the context of escalating antimicrobial resistance and increasing concern over environmentally mediated transmission of pathogens, such systems offer a scalable and sustainable adjunct to conventional infection prevention strategies.

Further research should evaluate efficacy against a broader range of clinically and industrially relevant microorganisms, including multidrug-resistant strains, biofilm communities, and spore-forming populations, as well as filamentous indoor moulds such as *Stachybotrys chartarum*, while also assessing long-term performance under more complex and realistic illumination and use conditions. Incorporating dark, temperature-matched controls, active heat-dissipation strategies, and spectrophotometric characterisation with defined media will further help isolate photochemical effects and decouple optical and nutritional influences. Together, these directions will strengthen the translational applicability of continuous aBL photoinactivation for indoor air and surface hygiene.

## Figures and Tables

**Table 1:** Irradiation and Dosage Values and Temperature Monitoring.

Distance (cm)	Irradiance (mW/cm^2^)	1 h Dosage (J/cm^2^)	3 h Dosage (J/cm^2^)	6 h Dosage (J/cm^2^)	24 h Dosage (J/cm^2^)
58	0.72	2.59	7.78	15.56	62.23

**Table 2:** Measures of central tendency for average temperature monitoring for the different experiments.

Metric	aBL LED Source	Fluorescent Light
Range (max)	39.6	32.5
Range (min)	28.6	26.3
Mean	35.8	28.1
±Standard deviation (*n* = 31,912)	0.9	0.8

**Table 3:** LSD post hoc groupings of treatments within each surface (*p* < 0.05).

Surface	Time (h)	EC–Fluor	BC–Fluor	EC–LED	BC–LED	F-Value	*p*-Value	η^2^
Agar	0	A	A	A	A	0.00	1.000	0.000
Agar	1–24	A	A	B	B	93.01–852.81	<0.001	0.978–0.996
Broth	0	A	A	A	A	0.00	1.000	0.000
Broth	1–24	A	A	B	C	93.01–852.81	<0.001	0.978–0.996
Glass	0	A	A	A	A	0.00	1.000	0.000
Glass	1–24	A	B	C	D	93.01–852.81	<0.001	0.978–0.996
Steel	0	A	A	A	A	0.00	1.000	0.000
Steel	1–24	A	B	C	D	93.01–852.81	<0.001	0.978–0.996

Abbreviations: EC = *E. coli*, BC = *B. cereus*, Fluor = Fluorescent. Treatments sharing a letter within a row are not significantly different (one-way ANOVA with LSD post hoc test, α = 0.05). Statistical analyses were performed on log_10_-transformed CFU data. F-values and corresponding *p*-values are shown for each timepoint; values for 1–24 h represent the range observed across timepoints. Effect sizes (η^2^) were large across all post-treatment timepoints, consistent with strong treatment effects.

## Data Availability

The original contributions presented in the study are included in the article, further inquiries can be directed to the corresponding authors.
